# Supplementary material for: Factors influencing user decision of telemedicine applications in Thailand
Source: PLoS One. 2025 Jun 4;20(6):e0325512. doi: 10.1371/journal.pone.0325512 (PMC12136430; doi:10.1371/journal.pone.0325512)
Supplement: S1 Table — (DOCX) [file pone.0325512.s001.docx]

**S1 Table. Reliability Test of Survey Data**

| **Variables** | | **Number of items** | **Cronbach's Alpha Coefficient** |
| --- | --- | --- | --- |
| **Unified theory of acceptance and use of technology (UTAUT2 Framework)** | | 25 | 0.968 |
|  | Performance Expectancy | 4 | 0.810 |
|  | Effort Expectancy | 5 | 0.852 |
|  | Social Influence | 4 | 0.854 |
|  | Facilitating Conditions | 3 | 0.770 |
|  | Hedonic Motivation | 3 | 0.813 |
|  | Price Value | 3 | 0.902 |
|  | Habit | 3 | 0.804 |
| **Information systems (IS) success model** | | 25 | 0.970 |
|  | System Quality | 5 | 0.862 |
|  | Information Quality | 5 | 0.868 |
|  | Service Quality | 5 | 0.892 |
|  | Use | 3 | 0.763 |
|  | User Satisfaction | 4 | 0.920 |
|  | Net Benefits | 3 | 0.808 |
| **Trust** | | 6 | 0.948 |
| **Perceived Risk** | | 6 | 0.917 |
| **Factors influencing the decision to use telemedicine application services** | | 4 | 0.991 |
