# Supplementary material for: Factors influencing user decision of telemedicine applications in Thailand
Source: PLoS One. 2025 Jun 4;20(6):e0325512. doi: 10.1371/journal.pone.0325512 (PMC12136430; doi:10.1371/journal.pone.0325512)
Supplement: S1 File — (PDF) [file pone.0325512.s002.pdf]

S1 File. The questionnaire in the English version

Questionnaire

Factors Influencing User Decision of Telemedicine Applications in Thailand

Screening Questions

Please put a checkmark ✓ in the box ❑ for the statement that best fits you (Choose only one)

1. Are you between the ages of 18 – 65?

❑ Yes

❑ No (End of Questionnaire)

2. Have you ever used telemedicine applications in Thailand?

❑ Yes

❑ No

Part 1 General Information of Respondents

Please put a checkmark ✓ in the box ❑ for the statement that best fits you (Choose only one)

1. Age

❑ 18 – 20 years old ❑ 21 – 30 years old ❑ 31 – 40 years old

❑ 41 – 50 years old ❑ 51 – 60 years old ❑ 61 years old and above

2. Sex

❑ Male ❑ Female

3. Education Level

❑ Below Bachelor's Degree ❑ Bachelor's Degree ❑ Postgraduate degree

4. Income (Monthly)

❑ < 10,000 Baht ❑ 10,000 - 20,000 Baht

❑ 20,001 – 30,000 Baht ❑ > 30,000 baht

5. Regions

❑ Bangkok ❑ Central Region (excluding Bangkok)

❑ Northern Region ❑ Northeastern Region

❑ Southern Region ❑ Eastern Region

6. Physical condition

❑ No underlying diseases. ❑ Have a medical condition.

Part 2: Questionnaire on the behavior of using telemedicine applications

Please mark ✓ into ❑ The text page that best matches your reality, just one answer for those who ever used telemedicine application.

1. Approximate frequency of using telemedicine applications

❑ 1 time per week ❑ Once a month ❑ 2-4 times a month.

❑ Once a year ❑ 2-5 times a year. ❑ More than 5 times a year.

❑ Once every few years.

2. Average cost of using telemedicine applications per visit

❑ Less than 500 Baht ❑ 500 – 1,000 Baht

❑ 1,001 – 2,000 Baht ❑ 2,001 – 3,000 Baht

❑ 3,001 – 4,000 Baht ❑ 4,001 – 5,000 Baht

❑ 5,001 Baht and above

3. The most common reasons for using telemedicine applications

❑ Health Promotion ❑ Treatment of Diseases

❑ Exercise ❑ Leisure

❑ Health Check-up ❑ Other (specified) ....

4. The Person Involved in Decision-Making for Using Telemedicine Application Services.

❑ Themselves ❑ Friends

❑ Family Members ❑ Supervisor/Organization/Company

❑ Salesperson ❑ Other (specified) ....

5. Source for information about Telemedicine Applications

❑ Print Media (Books, Magazine, Newspapers) ❑ Social Media

❑ Websites ❑ Radio/TV

❑ Other (specify) ....

Part 3: Questionnaire on Factors of Acceptance and Use of Integrated Technology 2 (Theory of Acceptance and Use of Technology: UTAUT2)

Please put a checkmark ✓ in the table that best fits your opinion on the statement (Choose only one)

Each score corresponds to a level of agreement as follows:

5 = strongly agree, 4=agree, 3=neutral, 2= disagree, 1= strongly disagree

Questions	Opinion				
	Strongly agree (5)	Agree (4)	Neutral (3)	Disagree (2)	Strongly Disagree (1)
**1. Performance Expectation**					
1.1 The use of telemedicine application allows you to receive information on health problems.					
1.2 You understand the format for using the telemedicine application.					
1.3 Using telemedicine application allows you to understand your own health problems.					
1.4 Using the telemedicine application saves you time to visit a health facility.					
**2. Effort Expectation**					
2.2 The telemedicine applications is easy to use.					
2.2 The operation of the telemedicine application is not complicated.					
2.3 You can use the telemedicine application very well.					
2.4 Telemedicine application has notifications					
2.5 Telemedicine application has a follow-up session.					
**3. Social Influence**					
3.1 Using telemedicine applications allows information to be exchanged through social networks.					
3.2 Telemedicine applications are recommended by healthcare professionals.					

Questions	Opinion				
	Strongly agree (5)	Agree (4)	Neutral (3)	Disagree (2)	Strongly Disagree (1)
3.3 Using telemedicine applications give you a good image.					
3.4 Telemedicine applications enhance knowledge about health.					
**4. Facilitating Conditions**					
4.1 You are able to navigate when using the telemedicine application.					
4.2 Telemedicine applications offer multiple device compatibility.					
4.3 The telemedicine applications align with your health needs.					
**5. Hedonic Motivation**					
5.1 You enjoy using the Telemedicine Application.					
5.2 You are satisfied with the use of the Telemedicine Application.					
5.3 You feel happy when you use the Telemedicine Application.					
**6. Price Value**					
6.1 Low cost of using telemedicine applications.					
6.2 Using telemedicine applications is cost-effective.					
6.3 Telemedicine Applications offer free subscription option.					
**7. Habit**					
7.1 You are familiar with using the telemedicine application for free.					
7.2 You frequently use the telemedicine application.					

Questions	Opinion				
	Strongly agree (5)	Agree (4)	Neutral (3)	Disagree (2)	Strongly Disagree (1)
7.3 When health problems arise, telemedicine is the first choice for care.					

Part 4: Questionnaire on Success Factors in the Use of Information Systems (IS Success Model)

Please put a checkmark ✓ in the table that best fits your opinion on the statement (Choose only one)

Each score corresponds to a level of agreement as follows:

5 = strongly agree, 4=agree, 3=neutral, 2= disagree, 1= strongly disagree

Questions	Opinion				
	**Strongly agree (5)**	**Agree (4)**	**Neutral (3)**	**Disagree (2)**	**Strongly Disagree (1)**
**1. System Quality**					
1.1 Telemedicine applications are easy to use.					
1.2 Telemedicine application system are stable.					
1.3 Telemedicine applications are responsive					
1.4 Telemedicine applications are readily accessible					
1.5 Telemedicine applications are convenient and can be used anytime, anywhere.					
**2. Information Quality**					
2.1 Information from telemedicine applications are complete and accurate.					
2.2 Information from telemedicine applications are easy to understand.					
2.3 The information in the telemedicine application is personalized.					
2.4 The information in the telemedicine application is secure.					
2.5 The data transmission of telemedicine applications is fast.					
**3. Service Quality**					
3.1 Satisfied after using the telemedicine application service.					
3.2 Establish a good relationship between telemedicine applications and the end users.					

Questions	Opinion				
	Strongly agree (5)	Agree (4)	Neutral (3)	Disagree (2)	Strongly Disagree (1)
3.3 Telemedicine doctors are attentive and hospitable.					
3.4 Telemedicine doctors are willing to provide service.					
3.5 Telemedicine doctors are very helpful in times of need.					
**4. Usage**					
4.1 You use telemedicine application regularly when you have health problems.					
4.2 Telemedicine applications have a good understanding of your health problems.					
4.3 Telemedicine applications are stable during use.					
**5. User satisfaction**					
5.1 You will continue to use the telemedicine application if you have health problems.					
5.2 You prefer using telemedicine applications.					
5.3 You wish to become or remain a member of the telemedicine application					
5.4 You will recommend the telemedicine application service to others.					
**6. Net Benefits**					
6.1 Using telemedicine application services is cost-effective.					
6.2 Using telemedicine application services saves travel time					
6.3 The use of the Telemedicine Application Service addresses your health issues..					

Part 5 Trust Factor Questionnaire

Please put a checkmark ✓ in the table that best fits your opinion on the statement (Choose only one)

Each score corresponds to a level of agreement as follows:

5 = strongly agree, 4=agree, 3=neutral, 2= disagree, 1= strongly disagree

**Questions**	**Opinion**				
	**Strongly agree (5)**	**Agree (4)**	**Neutral (3)**	**Disagree (2)**	**Strongly Disagree (1)**
**1. Trust**					
1.1 The application is reliable					
1.2 Believed that the use of telemedicine applications can meet health needs.					
1.3 When you use the telemedicine application, you feel reassured.					
1.4 You have a positive feeling about the counselling or answers provided in the telemedicine application.					
1.5 You find the telemedicine application convenient to use.					
1.6 You get fast response from using Telemedicine applications					

Part 6: Questionnaire on Perceived Risk Factors

Please put a checkmark ✓ in the table that best fits your opinion on the statement (Choose only one)

Each score corresponds to a level of agreement as follows:

5 = strongly agree, 4=agree, 3=neutral, 2= disagree, 1= strongly disagree

Questions	Opinion				
	**Strongly agree (5)**	**Agree (4)**	**Neutral (3)**	**Disagree (2)**	**Strongly Disagree (1)**
**1. Perceived Risk**					
1.1 Financial transactions in telemedicine applications are not secure.					
1.2 The system operation in telemedicine applications is incorrect.					
1.3 You cannot use the Telemedicine Application as expected.					
1.4 It is not worth purchasing services in telemedicine applications.					
1.5 Using the telemedicine application may cause your personal information to be leaked to other sources.					
1.6 Feel that installing and using telemedicine applications is a waste of time.					

Part 7: Questionnaire on the decision to use telemedicine application services in Thailand

Please put a checkmark ✓ in the table that best fits your opinion on the statement (Choose only one)

Each score corresponds to a level of agreement as follows:

5 = strongly agree, 4=agree, 3=neutral, 2= disagree, 1= strongly disagree

Questions	Opinion				
	Strongly agree (5)	Agree (4)	Strongly agree (5)	Disagree (2)	Strongly agree (5)
**Decision to use telemedicine application services**					
1.1 You intend to learn about the telemedicine application.					
1.2 You are interested in how telemedicine applications work.					
1.3 You believe that telemedicine applications can help solve health problems.					
1.4 You want to use the telemedicine application.					

Section 8 Comments and Suggestions

……………………………………………………………………………………………………..

……………………………………………………………………………………………………..

……………………………………………………………………………………………………..

……………………………………………………………………………………………………..

* Please take the time to answer the questionnaire*
