# Supplementary material for: Factors influencing user decision of telemedicine applications in Thailand
Source: PLoS One. 2025 Jun 4;20(6):e0325512. doi: 10.1371/journal.pone.0325512 (PMC12136430; doi:10.1371/journal.pone.0325512)
Supplement: S2 Table — (DOCX) [file pone.0325512.s003.docx]

**S2 Table. Kaiser-Meyer-Olkin Measure and Bartlett’s Test of 62 variables**

| Kaiser-Meyer-Olkin Measure of Sampling Adequacy. | | 0.952 |
| --- | --- | --- |
| Bartlett's Test of Sphericity | Approx. Chi-Square | 31236.064 |
|  | Degree of freedom | 1,891 |
|  | P-value for significance | <0.0001 |
