# Supplementary material for: Factors influencing user decision of telemedicine applications in Thailand
Source: PLoS One. 2025 Jun 4;20(6):e0325512. doi: 10.1371/journal.pone.0325512 (PMC12136430; doi:10.1371/journal.pone.0325512)
Supplement: S3 Table — (DOCX) [file pone.0325512.s004.docx]

**S3 Table. Principal Component Analysis (PCA)**

| Component | Initial Eigenvalues | | | Extraction Sums of Squared Loadings | | | Rotation Sums of Squared Loadings | | |
| --- | --- | --- | --- | --- | --- | --- | --- | --- | --- |
|  | Total | % of Variance | Cumulative % | Total | % of Variance | Cumulative % | Total | % of Variance | Cumulative % |
| 1 | 35.681 | 57.550 | 57.550 | 35.681 | 57.550 | 57.550 | 17.835 | 28.766 | 28.766 |
| 2 | 4.639 | 7.483 | 65.032 | 4.639 | 7.483 | 65.032 | 9.752 | 15.728 | 44.494 |
| 3 | 3.386 | 5.462 | 70.494 | 3.386 | 5.462 | 70.494 | 9.668 | 15.594 | 60.089 |
| 4 | 1.512 | 2.439 | 72.933 | 1.512 | 2.439 | 72.933 | 4.540 | 7.322 | 67.411 |
| 5 | 1.192 | 1.922 | 74.856 | 1.192 | 1.922 | 74.856 | 3.010 | 4.854 | 72.265 |
| 6 | 1.004 | 1.619 | 76.475 | 1.004 | 1.619 | 76.475 | 2.610 | 4.210 | **76.475** |
| 7 | 0.713 | 1.150 | 77.625 |  |  |  |  |  |  |
| 8 | 0.690 | 1.114 | 78.739 |  |  |  |  |  |  |
| 9 | 0.679 | 1.095 | 79.834 |  |  |  |  |  |  |
| 10 | 0.650 | 1.049 | 80.882 |  |  |  |  |  |  |
| 11 | 0.599 | 0.967 | 81.849 |  |  |  |  |  |  |
| 12 | 0.574 | 0.926 | 82.775 |  |  |  |  |  |  |
| 13 | 0.525 | 0.847 | 83.622 |  |  |  |  |  |  |
