# Supplementary material for: Factors influencing user decision of telemedicine applications in Thailand
Source: PLoS One. 2025 Jun 4;20(6):e0325512. doi: 10.1371/journal.pone.0325512 (PMC12136430; doi:10.1371/journal.pone.0325512)
Supplement: S4 Table — (DOCX) [file pone.0325512.s005.docx]

**S4 Table. Factor loading using Varimax rotation**

| **variable** | **Component** | | | | | |
| --- | --- | --- | --- | --- | --- | --- |
|  | **1** | **2** | **3** | **4** | **5** | **6** |
| You find the telemedicine application convenient to use. | 0.790 | 0.208 | 0.237 | 0.207 | 0.096 | 0.068 |
| Using telemedicine application services does not cause personal information to be leaked to other sources. | 0.789 | 0.231 | 0.243 | 0.119 | 0.146 | 0.099 |
| The application is reliable. | 0.785 | 0.200 | 0.207 | 0.172 | 0.133 | 0.141 |
| Feel that installing and using telemedicine applications is not a waste of time. | 0.781 | 0.218 | 0.269 | 0.210 | 0.026 | 0.083 |
| When you use the telemedicine application, you feel reassured. | 0.781 | 0.208 | 0.266 | 0.147 | 0.093 | 0.056 |
| Use telemedicine application regularly when you have health problems. | 0.780 | 0.199 | 0.258 | 0.204 | 0.089 | 0.072 |
| You frequently use the telemedicine application. | 0.779 | 0.273 | 0.266 | 0.144 | 0.108 | 0.031 |
| Will continue to use the telemedicine application if you have health problems. | 0.776 | 0.233 | 0.233 | 0.150 | 0.097 | 0.098 |
| Telemedicine applications align with health needs. | 0.776 | 0.207 | 0.270 | 0.171 | 0.112 | 0.092 |
| Will recommend the telemedicine application service to others | 0.774 | 0.224 | 0.242 | 0.154 | 0.110 | 0.156 |
| Prefer using telemedicine applications. | 0.771 | 0.206 | 0.199 | 0.186 | 0.168 | 0.187 |
| Have a positive feeling about the counseling or answers provided in the telemedicine application. | 0.766 | 0.207 | 0.258 | 0.174 | 0.080 | 0.096 |
| Feel that financial transactions in telemedicine applications are secure. | 0.764 | 0.243 | 0.262 | 0.113 | 0.123 | 0.103 |
| Believed that the use of telemedicine applications can meet health needs. | 0.759 | 0.178 | 0.223 | 0.191 | 0.136 | 0.158 |
| You get fast response from using Telemedicine applications | 0.759 | 0.226 | 0.235 | 0.177 | 0.257 | 0.079 |
| Satisfied with the use of the Telemedicine Application. | 0.757 | 0.257 | 0.274 | 0.124 | 0.101 | 0.084 |
| You wish to become or remain a member of the telemedicine application | 0.750 | 0.251 | 0.304 | 0.133 | 0.045 | 0.092 |
| The information in the telemedicine application is secure. | 0.748 | 0.248 | 0.337 | 0.128 | 0.172 | 0.062 |
| The information in the telemedicine application is personalized. | 0.748 | 0.222 | 0.265 | 0.185 | 0.143 | 0.045 |
| Telemedicine applications are recommended by healthcare professionals. | 0.748 | 0.252 | 0.271 | 0.114 | 0.152 | 0.134 |
| Feel happy when you use the Telemedicine Application. | 0.747 | 0.240 | 0.266 | 0.136 | 0.097 | 0.117 |
| Satisfied after using the telemedicine application service. | 0.746 | 0.260 | 0.272 | 0.182 | 0.110 | 0.057 |
| Telemedicine application services can be used as expected. | 0.735 | 0.197 | 0.231 | 0.110 | 0.222 | 0.187 |
| When health problems arise, telemedicine is the first choice for care. | 0.724 | 0.248 | 0.291 | 0.185 | 0.197 | 0.012 |
| Telemedicine applications are convenient and can be used anytime, anywhere. | 0.260 | 0.817 | 0.182 | 0.152 | 0.071 | 0.071 |
| Familiar with using the telemedicine application for free. | 0.284 | 0.805 | 0.185 | 0.060 | 0.080 | 0.159 |
| Can navigate when using the telemedicine application. | 0.244 | 0.793 | 0.155 | 0.150 | 0.121 | 0.137 |
| Operation of the telemedicine application is not complicated. | 0.254 | 0.790 | 0.142 | 0.225 | 0.130 | 0.116 |
| Can use the telemedicine application very well. | 0.257 | 0.787 | 0.154 | 0.208 | 0.000 | 0.114 |
| Telemedicine applications are easy to use. | 0.272 | 0.780 | 0.149 | 0.185 | 0.098 | 0.096 |
| You understand the format for using the telemedicine application. | 0.277 | 0.776 | 0.156 | 0.184 | 0.142 | 0.078 |
| The telemedicine application is easy to use. | 0.250 | 0.767 | 0.175 | 0.154 | 0.182 | 0.139 |
| Information from telemedicine applications are easy to understand. | 0.265 | 0.762 | 0.162 | 0.136 | 0.132 | 0.160 |
| Telemedicine applications offer multiple device compatibility. | 0.284 | 0.759 | 0.171 | 0.219 | 0.016 | 0.055 |
| Information from telemedicine applications are complete and accurate. | 0.340 | 0.190 | 0.763 | 0.164 | 0.152 | 0.052 |
| Telemedicine application has notifications. | 0.319 | 0.214 | 0.747 | 0.175 | 0.150 | 0.069 |
| Telemedicine applications are responsive | 0.406 | 0.175 | 0.736 | 0.210 | 0.092 | 0.062 |
| Telemedicine application system are stable. | 0.340 | 0.211 | 0.734 | 0.164 | 0.145 | 0.123 |
| The data transmission of telemedicine applications is fast. | 0.352 | 0.181 | 0.729 | 0.136 | 0.129 | 0.130 |
| Telemedicine application has a follow-up session. | 0.365 | 0.163 | 0.726 | 0.175 | 0.161 | 0.080 |
| Telemedicine applications are readily accessible | 0.333 | 0.194 | 0.723 | 0.175 | 0.148 | 0.127 |
| Using telemedicine applications allows information to be exchanged through social networks. | 0.348 | 0.176 | 0.721 | 0.172 | 0.190 | 0.161 |
| The use of telemedicine application allows you to receive information on health problems. | 0.356 | 0.165 | 0.707 | 0.132 | 0.145 | 0.224 |
| Telemedicine applications are stable during use. | 0.351 | 0.200 | 0.690 | 0.172 | 0.168 | 0.203 |
| The system operation in telemedicine applications is correct. | 0.411 | 0.152 | 0.673 | 0.176 | 0.159 | 0.191 |
| Enjoy using the Telemedicine Application. | 0.353 | 0.351 | 0.230 | 0.703 | 0.145 | 0.076 |
| Using telemedicine application services saves travel time. | 0.339 | 0.346 | 0.297 | 0.665 | 0.081 | 0.135 |
| It makes them understand their own health problems. | 0.342 | 0.322 | 0.244 | 0.657 | 0.100 | 0.179 |
| Using telemedicine applications gives a good image. | 0.326 | 0.339 | 0.265 | 0.639 | 0.151 | 0.203 |
| Telemedicine applications enhance knowledge about health. | 0.306 | 0.332 | 0.281 | 0.629 | 0.240 | 0.143 |
| Using the telemedicine application saves you time to visit a health facility. | 0.320 | 0.359 | 0.269 | 0.618 | 0.102 | 0.159 |
| The use of the telemedicine application service addresses your health issues.. | 0.314 | 0.363 | 0.388 | 0.538 | 0.031 | 0.174 |
| Using telemedicine applications is cost-effective. | 0.319 | 0.229 | 0.405 | 0.152 | 0.660 | 0.176 |
| Free telemedicine application subscription | 0.357 | 0.220 | 0.426 | 0.189 | 0.652 | 0.106 |
| Using telemedicine application services is cost-effective. | 0.387 | 0.205 | 0.391 | 0.213 | 0.635 | 0.089 |
| It is worth purchasing services in telemedicine applications. | 0.404 | 0.241 | 0.430 | 0.181 | 0.601 | 0.074 |
| Low cost of using telemedicine applications. | 0.368 | 0.227 | 0.466 | 0.087 | 0.576 | 0.197 |
| Telemedicine doctors are willing to provide service. | 0.253 | 0.417 | 0.352 | 0.229 | 0.090 | 0.634 |
| Telemedicine applications have a good understanding of your health problems. | 0.224 | 0.419 | 0.334 | 0.260 | 0.164 | 0.607 |
| Telemedicine doctors are attentive and hospitable. | 0.225 | 0.460 | 0.344 | 0.212 | 0.113 | 0.591 |
| Establish a good relationship between telemedicine applications and the end users. | 0.253 | 0.384 | 0.319 | 0.261 | 0.162 | 0.572 |
| Telemedicine doctors are very helpful in times of need. | 0.233 | 0.445 | 0.334 | 0.267 | 0.186 | 0.517 |
